# Omics Approaches for Identifying Physiological Adaptations to Genome Instability in Aging

**DOI:** 10.3390/ijms18112329

**Published:** 2017-11-04

**Authors:** Diletta Edifizi, Björn Schumacher

**Affiliations:** 1 Institute for Genome Stability in Ageing and Disease, Medical Faculty, University of Cologne, Joseph-Stelzmann-Str. 26, 50931 Cologne, Germany; didyedi@gmail.com; 2 Cologne Excellence Cluster for Cellular Stress Responses in Ageing-Associated Diseases (CECAD) and Systems Biology of Ageing Cologne, University of Cologne, Joseph-Stelzmann-Str. 26, 50931 Cologne, Germany

**Keywords:** DNA damage, aging, Nucleotide-excision repair (NER), Ultraviolet light (UV), Cockayne syndrome (CS), Xeroderma Pigmentosum (XP), growth hormone/insulin-like growth factor 1 (GH/IGF1) signaling, autophagy, protein homeostasis, lipid metabolism

## Abstract

DNA damage causally contributes to aging and age-related diseases. The declining functioning of tissues and organs during aging can lead to the increased risk of succumbing to aging-associated diseases. Congenital syndromes that are caused by heritable mutations in DNA repair pathways lead to cancer susceptibility and accelerated aging, thus underlining the importance of genome maintenance for withstanding aging. High-throughput mass-spectrometry-based approaches have recently contributed to identifying signalling response networks and gaining a more comprehensive understanding of the physiological adaptations occurring upon unrepaired DNA damage. The insulin-like signalling pathway has been implicated in a DNA damage response (DDR) network that includes epidermal growth factor (EGF)-, AMP-activated protein kinases (AMPK)- and the target of rapamycin (TOR)-like signalling pathways, which are known regulators of growth, metabolism, and stress responses. The same pathways, together with the autophagy-mediated proteostatic response and the decline in energy metabolism have also been found to be similarly regulated during natural aging, suggesting striking parallels in the physiological adaptation upon persistent DNA damage due to DNA repair defects and long-term low-level DNA damage accumulation occurring during natural aging. These insights will be an important starting point to study the interplay between signalling networks involved in progeroid syndromes that are caused by DNA repair deficiencies and to gain new understanding of the consequences of DNA damage in the aging process.

## 1. Introduction

Genome maintenance is important throughout life to counteract the accumulation of DNA damage. Unrepaired DNA damage can have a range of consequences including cell cycle arrest and senescence, apoptosis, cellular dysfunction and the accumulation of mutations. The causal role of DNA damage not only in cancer development but also in aging-associated diseases in general has been increasingly recognized in recent years. DNA lesions are constantly formed amid genotoxic attacks by exogenous sources such as ultraviolet light (UV) light and ionizing radiation (IR) or endogenous insults, such as reactive oxygen species (ROS) and metabolic byproducts. To overcome the potential deleterious effects of DNA damage accumulation, cells have evolved specialized DNA repair systems, each repairing specific types of lesions. Base excision repair (BER) rapidly removes ROS and oxidized bases produced during metabolic processes [1]. Mismatch repair (MMR) corrects mistakes missed by the replication machinery, through scanning the newly replicated strand [2]. The error-prone non-homologous end joining (NHEJ) [3] and the accurate homologous recombination (HR) pathways [4] are key mechanisms for repairing DNA double strand breaks (DSBs). Bulky DNA lesions that disturb the normal double-helical structure of DNA, such as UV-induced 6-4 pyrimidine photoproducts (6-4PPs) [5] and cyclobutane pyrimidine dimers (CPDs) [6], are repaired by the nucleotide excision repair (NER) [7]. 

Despite these highly specialized DNA repair systems, some lesions might be overlooked and persist, while others might be converted into mutations thus increasing the cancer risk with aging [8]. Congenital syndromes that are caused by heritable mutations in NER genes exemplify particularly well the distinct mechanisms through which DNA damage fuels cancer development and promotes the aging process. While defects that primarily affect the global-genome (GG-) NER, which surveys the entire genome for helix-distorting lesions, lead to the skin cancer susceptibility syndrome Xeroderma pigmentosum (XP), defects primarily disabling transcription-coupled (TC-) NER lead to severe growth retardation and premature aging in Cockayne syndrome (CS) patients [9]. 

The pathological consequences of unrepaired DNA damage are complex and so is the cellular DNA damage response that orchestrates physiological adaptations [10] ranging from the modulation of signalling pathways [11,12,13,14] to metabolic adjustments [15,16]. Interestingly, similar adaptations have been observed during aging [17,18] and upon stress conditions [19], suggesting that the aging organism responds to the accumulation of DNA lesions over time.

## 2. Adaptive Response to Stress

### 2.1 High-Throughput Approaches as a Tool to Identify Organismal Response Mechanisms upon Stress

**Figure 1:**
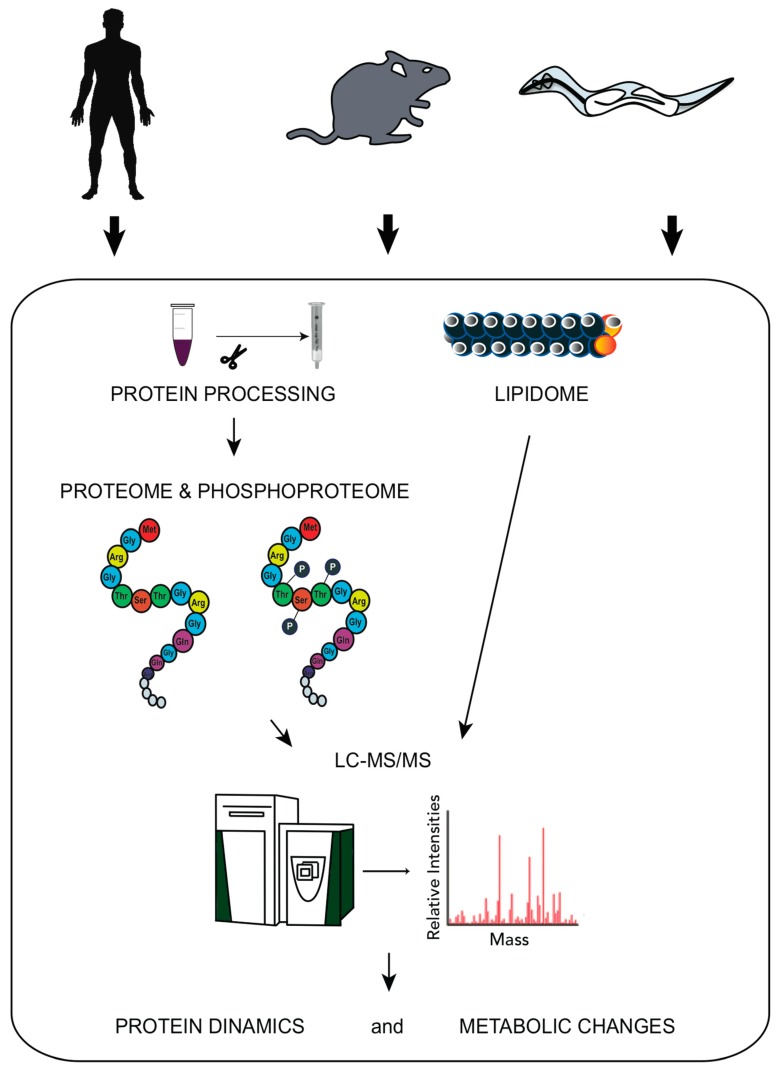
High throughput approaches applied to different model organisms. Large-scale experimental analysis allows the identification of global protein dynamics and metabolic changes that provide more insights into the full range of physiological adaptations upon normal and altered conditions. LC-MS/MS, liquid chromatography tandem-mass spectrometry.

Technological advances in mass-spectrometry (MS)-based approaches have made large-scale protein as well as lipid and metabolite quantification accessible and usable for a growing community of scientists across various fields of the life sciences [20]. Such approaches, applied to different model organisms and coupled to global transcriptome studies [21,22,23], are recently emerging to provide insights into the global protein dynamics and alterations in the carbohydrate, amino acid, and lipid metabolism during the physiological adaptations to stress [10,15,19,24], as well as during aging [17,25,26] (Figure 1 and Table 1).

In the model eukaryote *Saccharomyces cerevisiae*, proteome studies upon treatments with DNA-damaging agents (methyl methanesulfonate (MMS), 4-nitroquinoline 1-oxide (4NQO), *tert*-Butyl hydroperoxide (t-BuOOH) and UV) have highlighted the nucleus and nuclear periphery as hot spots, suggesting that chromatin remodelling, together with nucleo-cytoplasmic transport of RNA and protein, are important targets for the stress response, as well as the macromolecular trafficking mechanism which is used to signal to the rest of the cell [27,28,29] (Table 1). The yeast *S. cerevisiae* has been also an interesting model in the context of toxicological studies, to understand the global organismal response mechanisms to different environmental pollutants, such as metals, fungicides and antimicrobials [30].

The nematode *Caenorhabditis elegans* is a versatile metazoan model organism to perform similar coupled omics and bioinformatics in vivo studies. Upon different conditions of heat, osmotic, and oxidative-stress [16,19], or after genotoxic UV-treatment in a background of NER deficiencies [10], many of the major cellular processes, such as chromatin remodeling, protein homeostasis and lipid metabolism were affected (Table 1). These stress response mechanisms, coupled to organismal metabolic changes, were also found to be similarly regulated in the nematode during aging [17,18,31], suggesting an active role of stresses and DNA damage accumulation in the physiological adaptations manifested in aged animals.

Coupled metabolomics and proteomics studies have also been performed in murine models, reporting an interesting readout, such as alterations at the level of lipid metabolism and macromolecular trafficking, including dynamic mechanisms of stress sensing [15,32,33,34] (Table 1). Protein refolding and degradation, as well as energy metabolism were also conserved response mechanisms in a murine model of aging [35,36]. The further application of these omics approaches within medical research in humans, opens new perspectives to the identification of biomarkers for organismal stress that are associated to aging [37,38], as well as a future of personalized treatments.

### 2.2 In Vivo Models to Study Adaptations to Nucleotide-Excision Repair (NER) Defects

Due to the highly complex phenotypes in human patients with congenital NER syndromes [39], corresponding mouse mutants have been generated to model the disease aetiology [40,41]. Transcriptome analysis performed in mouse mutants carrying similar genetic defects as human patients suffering from progeroid CS or the related XPF-ERCC1 progeria (XFE), have highlighted that, similarly to normative aging mice [11,42,43], there is a dampening of the growth hormone/insulin-like growth factor 1 (GH/IGF1)-mediated somatotropic axis [44,45], a conserved signalling pathway regulating development, stress resistance and longevity [46,47,48]. 

In worms as in mammals, the insulin/insulin-like growth factor signalling (IIS), a central component of the somatotropic axis, responds to transcription-blocking lesions, and through its effector, the transcription factor DAF-16 (*C. elegans* homologue of the FOXO family of transcription factors), elevates the tolerance to persistent DNA damage [12,49]. 

Due to the exquisitely complex physiological alterations occurring in the mouse models with DNA repair defects, particularly during developmental growth, the nematode *C. elegans* provides a relatively simple metazoan model to better understand the organismal consequences of unrepaired DNA damage and to study aging [18,31]. *C. elegans* has a well-defined developmental cycle and most of the major mammalian DNA repair pathways, including NER, are conserved to the molecular level [50]. Interestingly, in *C. elegans* the mutations in the two NER sub-pathways result in distinct outcomes when the worm is challenged with UV irradiation, reflecting the distinct human phenotypes of XP and CS. UV-treated GG-NER-deficient *xpc-1* animals display genome instability in proliferating cell types. In worms, most cell divisions occur during early embryonic development and in the germline throughout the animals’ life. Genome instability in proliferating cells is a root cause for cancer development in humans, thus emphasizing the model character for a causal event for skin cancer development in XP patients. In contrast, TC-NER- deficient *csb-1* or *csa-1* mutants arrest somatic developmental growth, and during adulthood somatic tissues degenerate upon UV exposure. CS patients display severe postnatal growth defects and premature aging underlining the worm’s model character for some important aspects of the human disease. The UV sensitivity of *csb-1* mutants can be enhanced when GG-NER is also defective, as in the case of completely NER-deficient *xpc-1*, *csb-1* double-mutants or *xpa-1,* indicating that the distinct NER initiating mechanisms can to some degree compensate for each other in line with the synthetic phenotypes of *Xpc* and *Csb* mutations in mice*.* Thus, a fundamental consequence of the distinct NER mutations are recapitulated in *C. elegans,* thus making the worm an interesting model to study the distinct in vivo responses to unrepaired DNA damage that are relevant for development, cancer, and aging in humans.

Transcriptome and proteome studies in *C. elegans*, have also contributed to the identification of the key regulators of stress responses [12,19,51,52,53] and longevity [10,17,31], with the conserved IIS pathway taking centre stage [17,54,55,56,57]. Similar to the somatotropic attenuation observed in NER mutant mice, in NER deficient *C. elegans,* the transcription factor DAF-16/FOXO, which is activated when IIS is attenuated, overcomes the developmental delay and elevates the tolerance to unrepaired DNA lesions [12,49,58].

### 2.3 The Response to Unrepaired DNA Damage upon Nucleotide Excision Repair (NER) Deficiencies Involves Mechanisms that Regulate the Aging Process

**Figure 2:**
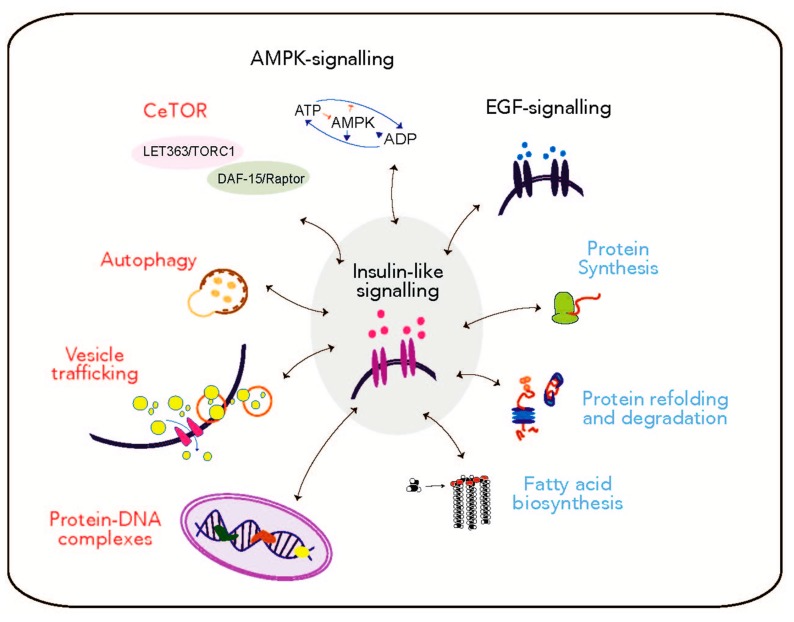
Map of differentially-regulated pathways in response to persistent DNA damage in nucleotide-excision repair (NER) deficient animals. Insulin-like signalling comprises a central node of a DNA damage response network, which involves the regulation of the epidermal growth factor (EGF)-, and AMP-activated protein kinase (AMPK)-like signalling pathways. The impaired proteostasis can lead to a general decrease in energy level, as exemplified by the attenuated fatty acid metabolism, and can be compensated by a shift towards autophagy. The processes that are downregulated or upregulated upon unrepaired DNA lesions are depicted in blue and red, respectively. CeTOR: *C. elegans* target of rapamycin; DAF, abnormal dauer formation.

A recent multidimensional omics analysis of the response to persistent DNA damage in an NER-deficient *C. elegans* model has highlighted the interaction of the IIS network with other evolutionarily-conserved signalling pathways, found previously to be implicated in the regulation of growth, metabolism, stress response and to be similarly regulated during aging [10,17,18]. In *C. elegans* mutants that lack the ability to remove UV-induced lesions due to mutations in the NER components *xpc-1* and *csb-1,* which are required for initiating GG-NER and TC-NER, respectively, have been used to investigate consequences of persistent DNA lesions. Using this paradigm of persistent DNA damage, the insulin-like growth factor-1 receptor (IGF-1R) homologue DAF-2, functioning as upstream component of the IIS signalling, has been identified as central hub of a network of UV-response genes [12] and proteins [10] that regulate larval development and longevity (Figure 2).

The combined assessment of proteome, lipidome, and phosphoproteome allows drawing a comprehensive picture of the interplay between the key signalling pathways that respond to altered conditions such as the persistence of DNA lesions (Figure 1 and Figure 2). 

One of the pathways regulated in response to persistent DNA damage is the AMP-activated protein kinase (AMPK)-like signalling, which plays a central role in controlling the organismal energy metabolism [59,60,61] upstream of the target of rapamycin (TOR) [10,13,62,63] (Figure 2). TOR signalling is involved in regulating translation and protein synthesis, autophagy [64,65,66,67], as well as longevity, under the influence of the IIS signalling [68,69]. The activity of the IIS and TOR pathways in controlling cell growth and survival is also affected by the epidermal growth factor (EGF) signalling [70,71], another central platform involved in controlling lipid metabolism through the cascade involving the phospholipase Cγ (PLC)/protein kinase C (PKC) [72], and found to be also regulated upon persistent DNA damage (Figure 2).

The physiological adaptations driven by these key signalling pathways in NER-deficient *C. elegans* mutants are consistent with previous reports from studies of aged animals [18,73,74], suggesting that the systemic metabolic responses observed upon acute DNA damage bear similarities with the adaptive physiological response upon long-term low-level DNA damage accumulation occurring during natural aging. 

A characteristic hallmark of aging that plays a role in aging-related neurodegenerative disease is the impairment of the proteostasis network [75,76,77], which represents a fundamental mechanism involved in maintaining cellular protein quality control. Proteome studies of aged *C. elegans* [17,19] and of NER-deficient mutants unable to repair the DNA damage [10] both revealed a dampening of protein homeostasis (Figure 2), indicative of an impairment in the clearing of aberrant proteins during both processes. Misfolded proteins failing to being properly refolded or degraded due to impaired protein homeostasis, are targeted for autophagic degradation, which could function as a compensatory response to clear them and recycle their component amino acids [78,79,80,81,82]. The accumulation of aberrant proteins during aging [83,84,85] and in age-related neurodegenerative disorders, such as Alzheimer’s disease (AD) [86], Parkinson’s disease (PD) [87] and Amyotrophic lateral sclerosis (ALS) [88] has been associated with an age-related decline in autophagic activity [64,89,90]. The idea supporting the contribution of dysfunctional autophagy to aging has been also reported in *C. elegans* studies, that show autophagy genes as being essential for lifespan extension [91], under the regulation of nutrient-sensing longevity processes as the IIS and TOR pathways [92,93,94]. 

Autophagy has emerged as a key player in modulating aging also by affecting lipid homeostasis [95]. Under unfavourable conditions, such as nutrient deprivation, the inhibition of the upstream regulator TOR [64,96] allows the activation of autophagy, which favours lipid mobilization to use them as energy source [97,98]. Similarly to observations in aging worms [17,18], the protein synthesis, refolding and degradation processes were also found to be impaired in *C. elegans* carrying persistent DNA damage due to the impaired NER machinery. In addition, energy levels appear to decrease, as indicated by a dampening of lipid metabolism, following genotoxic treatment (Figure 2). To counteract the accumulation of aberrant proteins and to promote the utilization of energy from lipid storage, autophagy is activated, potentially as a compensatory mechanism to withstand the unrepaired DNA damage [10,99]. The induction of autophagy and its association with changes in lipid metabolism have also been reported as mechanisms involved in metabolic responses in prematurely aging mice [73], reinforcing the concept that upon persistent accumulation of DNA lesions, animals establish a metabolic shift reminiscent of adaptations occurring during the natural aging process [11,18,26,31]. 

The accumulation of aberrant proteins with the consequent activation of autophagy are also connected with intra/extracellular vesicle trafficking, an important mechanism regulating neuronal functions [100], which has been reported as altered during aging and in neurodegenerative disorders such as AD and PD [101,102,103]. The pathophysiology of PD, for example, is characterized by an impaired axonal transport of autophagosomes due to the presence of oligomeric α-synuclein that alters synaptic vesicle distribution and intracellular neurotransmitter trafficking [104]. In contrast to the decrease of intracellular trafficking upon aging, in the *C. elegans* model of NER deficiency upon UV-irradiation, vesicle trafficking and synaptic transmission are promoted [10] (Figure 2), as indicated by the increased expression of members of the synaptic machinery and G protein-coupled receptor (GPCR) signalling, which play an essential role in neuronal communication [105]. This suggests that there might be a release of signals from genotoxically-compromised cells to potentially mediate the organismal adaptation to the unrepaired DNA damage. 

Chromatin remodelling and histone modifications, which regulate replication, transcription, and repair [106,107], play an important role in response to persistent DNA damage and during the aging process [10,108,109,110,111] (Figure 2). Alterations at the level of the epigenetic machinery have been seen to be involved in triggering modifications on the transcriptional level of the genes involved in the pathogenesis of age-related diseases such as AD and PD [112,113,114], characterized by the accumulation of misfolded and aggregated proteins and impaired proteostasis [115,116]. Moreover, epigenetic mechanisms and chromatin remodelling play an important role in influencing the IIS signalling effector DAF-16, to promote stress resistance and modulate longevity [12,117,118,119].

The integration of MS-based omics studies assessing proteins, post-translational modifications (PTMs), and metabolomics therefore provide insight into the physiological adaptations to genome instability in aging, aiding the deciphering of the hubs of the signalling networks and their interaction. Assessing the status of the network of response mechanisms to DNA damage could greatly advance the identification of potential targets for future therapeutic interventions for DNA-damage-driven aging-associated diseases.

## 3. Conclusions

In humans, mutations in NER genes lead to rare congenital disorders that are characterized by complex clinical phenotypes, ranging from elevated skin cancer susceptibility to growth retardation and premature aging. Important insights into the physiological consequences of NER mutations have been provided by studies of mouse mutants in various NER genes, which, although essential as disease models, exhibit rather complex phenotypes. The nematode *C. elegans* provides a greatly simplified model allowing the in vivo analysis of the responses to persistent DNA damage through large-scale mass-spectrometry (MS)-based studies. Nowadays, the technological advances of multiple omics MS-based approaches, allow the assessment of a large array of proteins and metabolites, important for identifying signal transduction networks responding to stress and aging. Each type of omics data typically provides important insight into the biological pathways that are differentially regulated upon specific stress conditions, but they should be combined to obtain a complete overview about disease causes and consequences. The integration of these multi-omics approaches, on model organisms and ultimately on humans, offer in fact an opportunity to unravel potential mechanisms causative of various diseases, as the mentioned progeroid syndromes, and to identify the physiological adaptations occurring in the aging process.

The identification, in *C. elegans* NER deficient mutants, of the IIS pathway as central node of a signalling network regulating growth, metabolism, and stress responses important during aging, suggest that adaptations to both acute and long-term low-level genome instability trigger a “survival response” that promotes the preservation of tissue functionality. 

## Figures and Tables

**Table 1:** Omics approaches applied to model organisms to identify the molecular mechanisms mostly involved upon stress conditions.

Model Organism	Stress Condition	Study	Affected Processes
*Saccharomyces cerevisiae*	DNA-damaging agents (MMS,4NQO,T-BUOOH and UV)	Begley et al. 2002 [27] Begley et al. 2004 [28] Said et al. 2004 [29]	Chromatin remodeling Nucleo-cytoplasmic transport of RNA and proteins Macromolecular trafficking Cytoskeleton remodeling Protein and Lipid metabolism
*Caenorhabditis elegans*	UV irradiation upon NER deficiency	Edifizi et al. 2017 [10]	Chromatin remodeling
			Protein homeostasis
			Protein refolding and degradation
			Macromolecular trafficking
			Fatty and amino acid metabolism
			Insulin-, EGF-, and AMPK-like signaling pathways
	Heat, osmotic, and oxidative-stress	Horikawa et al. 2009 [16] Liang et al. 2014 [19]	Fatty-acid metabolism Protein homeostasis
	Aging	Copes et al. 2015 [31] Walther et al. 2015 [17] Narayan et al. 2016 [18]	Fatty and amino acid metabolism Protein homeostasis Protein refolding and degradation Peroxisomal enzymes Insulin-like signaling pathway
*Mus musculus/ Rattus norvegicus*	Heat and chronic stress	Ippolito et al. 2014 [32]	Fatty and amino acid metabolism
		Oliveira et al 2015 [15]	
	Nutrient stress	Magliarelli et al. 2016 [33]	Post-translational modifications
			Macromolecular trafficking
	Copper oxide nanoparticles	Triboulet et al. 2015 [34]	Oxidative stress response
			Macrophage immune responses
	Aging	Chakravarti et al. 2009 [35] Stauch et al. 2015 [36]	Protein refolding and degradation Macromolecular trafficking Cellular metabolism
*Homo sapiens*	Nutrient stress coupled to physical exercise	Chorell et al. 2009 [37]	Fatty and amino acid metabolism
	Aging / aging-related diseses	Valdes et al. 2013 [38] Montoliou et al. 2014 [25]	Fatty and amino acid metabolism Oxidative stress response Protein refolding and degradation Macromolecular trafficking

## References

[1] Fortini P., Pascucci B., Parlanti E., D’Errico M., Simonelli V., Dogliotti E. (2003). The base excision repair: Mechanisms and its relevance for cancer susceptibility. Biochimie.

[2] Li G.-M. (2008). Mechanisms and functions of DNA mismatch repair. Cell Res..

[3] Lieber M.R. (2010). The mechanism of double-strand DNA break repair by the nonhomologous DNA end-joining pathway. Annu. Rev. Biochem..

[4] Sung P., Klein H. (2006). Mechanism of homologous recombination: Mediators and helicases take on regulatory functions. Nat. Rev. Mol. Cell Biol..

[5] Matsunaga T., Hieda K., Nikaido O. (1991). Wavelength dependent formation of thymine dimers and (6-4) photoproducts in DNA by monochromatic ultraviolet light ranging from 150 to 365 nm. Photochem. Photobiol..

[6] Setlow R.B., Carrier W.L. (1966). Pyrimidine dimers in ultraviolet-irradiated DNA’s. J. Mol. Biol..

[7] Cleaver J.E., Lam E.T., Revet I. (2009). Disorders of nucleotide excision repair: The genetic and molecular basis of heterogeneity. Nat. Rev. Genet..

[8] Vijg J., Suh Y. (2013). Genome Instability and Aging. Annu. Rev. Physiol..

[9] Edifizi D., Schumacher B. (2015). Genome Instability in Development and Aging: Insights from Nucleotide Excision Repair in Humans, Mice, and Worms. Biomolecules.

[10] Edifizi D., Nolte H., Babu V., Castells-Roca L., Mueller M.M., Brodesser S., Krüger M., Schumacher B. (2017). Multilayered Reprogramming in Response to Persistent DNA Damage in *C. elegans*. Cell Rep..

[11] Garinis G.A., Uittenboogaard L.M., Stachelscheid H., Fousteri M., van IJcken W., Breit T.M., van Steeg H., Mullenders L.H. F., van der Horst G.T. J., Brüning J.C. (2009). Persistent transcription-blocking DNA lesions trigger somatic growth attenuation associated with longevity. Nat. Cell Biol..

[12] Mueller M.M., Castells-Roca L., Babu V., Ermolaeva M.A., Muller R.-U., Frommolt P., Williams A.B., Greiss S., Schneider J.I., Benzing T. (2014). DAF-16/FOXO and EGL-27/GATA promote developmental growth in response to persistent somatic DNA damage. Nat. Cell Biol..

[13] Curtis R., O’Connor G., DiStefano P.S. (2006). Aging networks in *Caenorhabditis elegans*: AMP-activated protein kinase (aak-2) links multiple aging and metabolism pathways. Aging Cell.

[14] Wu C.L., Qiang L., Han W., Ming M., Viollet B., He Y.Y. (2013). Role of AMPK in UVB-induced DNA damage repair and growth control. Oncogene.

[15] Oliveira T.G., Chan R.B., Bravo F.V., Miranda A., Silva R.R., Zhou B., Marques F., Pinto V., Cerqueira J.J., di Paolo G. (2015). The impact of chronic stress on the rat brain lipidome. Mol. Psychiatry.

[16] Horikawa M., Sakamoto K. (2009). Fatty-acid metabolism is involved in stress-resistance mechanisms of *Caenorhabditis elegans*. Biochem. Biophys. Res. Commun..

[17] Walther D.M., Kasturi P., Zheng M., Pinkert S., Vecchi G., Ciryam P., Morimoto R.I., Dobson C.M., Vendruscolo M., Mann M. (2015). Widespread proteome remodeling and aggregation in aging *C. elegans*. Cell.

[18] Narayan V., Ly T., Pourkarimi E., Murillo A.B., Gartner A., Lamond A.I., Kenyon C. (2016). Deep proteome analysis identifies age-related processes in *C. elegans*. Cell Syst..

[19] Liang V., Ullrich M., Lam H., Chew Y.L., Banister S., Song X., Zaw T., Kassiou M., Götz J., Nicholas H.R. (2014). Altered proteostasis in aging and heat shock response in *C. elegans* revealed by analysis of the global and de novo synthesized proteome. Cell. Mol. Life Sci..

[20] Von Stechow L., Olsen J.V. (2017). Proteomics insights into DNA damage response and translating this knowledge to clinical strategies. Proteomics.

[21] Gunawardana Y., Niranjan M. (2013). Bridging the gap between transcriptome and proteome measurements identifies post-translationally regulated genes. Bioinformatics.

[22] Lackner D.H., Schmidt M.W., Wu S., Wolf D.A., Bähler J. (2012). Regulation of transcriptome, translation, and proteome in response to environmental stress in fission yeast. Genome Biol..

[23] Derks K.W.J., Hoeijmakers J.H.J., Pothof J. (2014). The DNA damage response: The omics era and its impact. DNA Repair.

[24] Hirai M.Y., Yano M., Goodenowe D.B., Kanaya S., Kimura T., Awazuhara M., Arita M., Fujiwara T., Saito K. (2004). Integration of transcriptomics and metabolomics for understanding of global responses to nutritional stresses in Arabidopsis thaliana. Proc. Natl. Acad. Sci. USA.

[25] Montoliu I., Scherer M., Beguelin F., DaSilva L., Mari D., Salvioli S., Martin F.-P.J., Capri M., Bucci L., Ostan R. (2014). Serum profiling of healthy aging identifies phospho- and sphingolipid species as markers of human longevity. Aging.

[26] Hou N.S., Taubert S. (2012). Function and Regulation of Lipid Biology in *Caenorhabditis elegans* Aging. Front Physiol..

[27] Begley T.J., Rosenbach A.S., Ideker T., Samson L.D. (2002). Damage recovery pathways in Saccharomyces cerevisiae revealed by genomic phenotyping and interactome mapping. Mol. Cancer Res..

[28] Begley T.J., Rosenbach A.S., Ideker T., Samson L.D. (2004). Hot spots for modulating toxicity identified by genomic phenotyping and localization mapping. Mol. Cell.

[29] Said M.R., Begley T.J., Oppenheim A.V., Lauffenburger D.A., Samson L.D. (2004). Global network analysis of phenotypic effects: Protein networks and toxicity modulation in *Saccharomyces cerevisiae*. Proc. Natl. Acad. Sci. USA.

[30] Dos Santos S.C., Teixeira M.C., Cabrito T.R., Sá-Correia I. (2012). Yeast toxicogenomics: Genome-wide responses to chemical stresses with impact in environmental health, pharmacology, and biotechnology. Front. Genet..

[31] Copes N., Edwards C., Chaput D., Saifee M., Barjuca I., Nelson D., Paraggio A., Saad P., Lipps D., Stevens S.M. (2015). Metabolome and proteome changes with aging in *Caenorhabditis elegans*. Exp. Gerontol..

[32] Ippolito D.L., Lewis J.A., Yu C., Leon L.R., Stallings J.D. (2014). Alteration in circulating metabolites during and after heat stress in the conscious rat: Potential biomarkers of exposure and organ-specific injury. BMC Physiol..

[33] De Fatima Magliarelli H., Matondo M., Mészáros G., Goginashvili A., Erbs E., Zhang Z., Mihlan M., Wolfrum C., Aebersold R., Sumara I. (2016). Liver ubiquitome uncovers nutrient-stress-mediated trafficking and secretion of complement C3. Cell Death Dis..

[34] Triboulet S., Aude-Garcia C., Armand L., Collin-Faure V., Chevallet M., Diemer H., Gerdil A., Proamer F., Strub J.-M., Habert A. (2015). Comparative proteomic analysis of the molecular responses of mouse macrophages to titanium dioxide and copper oxide nanoparticles unravels some toxic mechanisms for copper oxide nanoparticles in macrophages. PLoS ONE.

[35] Chakravarti B., Seshi B., Ratanaprayul W., Dalal N., Lin L., Raval A., Chakravarti D.N. (2009). Proteome profiling of aging in mouse models: Differential expression of proteins involved in metabolism, transport, and stress response in kidney. Proteomics.

[36] Stauch K.L., Purnell P.R., Villeneuve L.M., Fox H.S. (2015). Data for mitochondrial proteomic alterations in the aging mouse brain. Data Brief.

[37] Chorell E., Moritz T., Branth S., Antti H., Svensson M.B. (2009). Predictive metabolomics evaluation of nutrition-modulated metabolic stress responses in human blood serum during the early recovery phase of strenuous physical exercise. J. Proteome Res..

[38] Valdes A.M., Glass D., Spector T.D. (2013). Omics technologies and the study of human ageing. Nat. Rev. Genet..

[39] Schumacher B., Garinis G.A., Hoeijmakers J.H.J. (2008). Age to survive: DNA damage and aging. Trends Genet..

[40] Kamileri I., Karakasilioti I., Sideri A., Kosteas T., Tatarakis A., Talianidis I., Garinis G.A. (2012). Defective transcription initiation causes postnatal growth failure in a mouse model of nucleotide excision repair (NER) progeria. Proc. Natl. Acad. Sci. USA..

[41] Van de Ven M., Andressoo J.O., Holcomb V.B., von Lindern M., Jong W.M. C., de Zeeuw C.I., Suh Y., Hasty P., Hoeijmakers J.H.J., van der Horst G.T. J. (2006). Adaptive stress response in segmental progeria resembles long-lived dwarfism and calorie restriction in mice. PLoS Genet..

[42] Schumacher B., van der Pluijm I., Moorhouse M.J., Kosteas T., Robinson A.R., Suh Y., Breit T.M., van Steeg H., Niedernhofer L.J., van IJcken W. (2008). Delayed and Accelerated Aging Share Common Longevity Assurance Mechanisms. PLoS Genet..

[43] Carter C.S., Ramsey M.M., Sonntag W.E. (2002). A critical analysis of the role of growth hormone and IGF-1 in aging and lifespan. Trends Genet..

[44] Van der Pluijm I., Garinis G.A., Brandt R.M.C., Gorgels T.G.M.F., Wijnhoven S.W., Diderich K.E.M., de Wit J., Mitchell J.R., van Oostrom C., Beems R. (2006). Impaired Genome Maintenance Suppresses the Growth Hormone–Insulin-Like Growth Factor 1 Axis in Mice with Cockayne Syndrome. PLoS Biol..

[45] Niedernhofer L.J., Garinis G.A., Raams A., Lalai A.S., Robinson A.R., Appeldoorn E., Odijk H., Oostendorp R., Ahmad A., van Leeuwen W. (2006). A new progeroid syndrome reveals that genotoxic stress suppresses the somatotroph axis. Nature.

[46] Holzenberger M., Dupont J., Ducos B., Leneuve P., Géloën A., Even P.C., Cervera P., Le Bouc Y. (2003). IGF-1 receptor regulates lifespan and resistance to oxidative stress in mice. Nature.

[47] Junnila R.K., List E.O., Berryman D.E., Murrey J.W., Kopchick J.J. (2013). The GH/IGF-1 axis in ageing and longevity. Nat. Rev. Endocrinol..

[48] Bonkowski M.S., Pamenter R.W., Rocha J.S., Masternak M.M., Panici J.A., Bartke A. (2006). Long-lived growth hormone receptor knockout mice show a delay in age-related changes of body composition and bone characteristics. J. Gerontol. A Biol. Sci. Med. Sci..

[49] Castells-Roca L., Mueller M.M., Schumacher B. (2015). Longevity through DNA damage tolerance. Cell Cycle.

[50] Lans H., Vermeulen W. (2011). Nucleotide Excision Repair in *Caenorhabditis elegans*. Mol. Biol. Int..

[51] Larance M., Pourkarimi E., Wang B., Brenes Murillo A., Kent R., Lamond A.I., Gartner A. (2015). Global Proteomics Analysis of the Response to Starvation in *C. elegans*. Mol. Cell Proteom..

[52] Boyd W.A., Crocker T.L., Rodriguez A.M., Leung M.C.K., Wade Lehmann D., Freedman J.H., van Houten B., Meyer J.N. (2010). Nucleotide excision repair genes are expressed at low levels and are not detectably inducible in *Caenorhabditis elegans* somatic tissues, but their function is required for normal adult life after UVC exposure. Mutat. Res. Fundam. Mol. Mech. Mutagen..

[53] Pontoizeau C., Mouchiroud L., Molin L., Mergoud-dit-Lamarche A., Dallière N., Toulhoat P., Elena-Herrmann B., Solari F. (2014). Metabolomics Analysis Uncovers That Dietary Restriction Buffers Metabolic Changes Associated with Aging in *Caenorhabditis elegans*. J. Proteom. Res..

[54] McElwee J.J., Schuster E., Blanc E., Thomas J.H., Gems D. (2004). Shared transcriptional signature in *Caenorhabditis elegans* Dauer larvae and long-lived daf-2 mutants implicates detoxification system in longevity assurance. J. Biol. Chem..

[55] Golden T.R., Melov S. (2004). Microarray analysis of gene expression with age in individual nematodes. Aging Cell.

[56] Kenyon C., Chang J., Gensch E., Rudner A., Tabtiang R. (1993). A *C. elegans* mutant that lives twice as long as wild type. Nature.

[57] Ogg S., Paradis S., Gottlieb S., Patterson G.I., Lee L., Tissenbaum H.A., Ruvkun G. (1997). The Fork head transcription factor DAF-16 transduces insulin-like metabolic and longevity signals in *C. elegans*. Nature.

[58] Henderson S.T., Johnson T.E. (2001). daf-16 integrates developmental and environmental inputs to mediate aging in the nematode *Caenorhabditis elegans*. Curr. Biol..

[59] Mair W., Morantte I., Rodrigues A.P.C., Manning G., Montminy M., Shaw R.J., Dillin A. (2012). Lifespan extension induced by AMPK and calcineurin is mediated by CRTC-1 and CREB. Nature.

[60] Apfeld J., O’Connor G., McDonagh T., DiStefano P.S., Curtis R. (2004). The AMP-activated protein kinase AAK-2 links energy levels and insulin-like signals to lifespan in *C. elegans*. Genes Dev..

[61] Hardie D.G., Ross F.A., Hawley S.A. (2012). AMPK: A nutrient and energy sensor that maintains energy homeostasis. Nat. Rev. Mol. Cell Biol..

[62] Mihaylova M.M., Shaw R.J. (2011). The AMPK signalling pathway coordinates cell growth, autophagy and metabolism. Nat. Cell Biol..

[63] Xu J., Ji J., Yan X.-H. (2012). Cross-talk between AMPK and mTOR in regulating energy balance. Crit. Rev. Food Sci. Nutr..

[64] Rubinsztein D.C., Mariño G., Kroemer G. (2011). Autophagy and Aging. Cell.

[65] Ravikumar B., Sarkar S., Davies J.E., Futter M., Garcia-Arencibia M., Green-Thompson Z.W., Jimenez-Sanchez M., Korolchuk V.I., Lichtenberg M., Luo S. (2010). Regulation of mammalian autophagy in physiology and pathophysiology. Physiol. Rev..

[66] Wullschleger S., Loewith R., Hall M.N. (2006). TOR signaling in growth and metabolism. Cell.

[67] Kim J.E., Chen J. (2004). Regulation of peroxisome proliferator-activated receptor-γ activity by mammalian target of rapamycin and amino acids in adipogenesis. Diabetes.

[68] Hay N. (2011). Interplay between FOXO, TOR, and Akt. Biochim. Biophys. Acta.

[69] Johnson S.C., Rabinovitch P.S., Kaeberlein M. (2013). mTOR is a key modulator of ageing and age-related disease. Nature.

[70] Iwasa H., Yu S., Xue J., Driscoll M. (2010). Novel EGF pathway regulators modulate *C. elegans* healthspan and lifespan via EGF receptor, PLC-γ, and IP3R activation. Aging Cell.

[71] Liu G., Rogers J., Murphy C.T., Rongo C. (2011). EGF signalling activates the ubiquitin proteasome system to modulate *C. elegans* lifespan. EMBO J..

[72] Jorissen R.N., Walker F., Pouliot N., Garrett T.P.J., Ward C.W., Burgess A.W. (2003). Epidermal growth factor receptor: mechanisms of activation and signalling. Exp. Cell Res..

[73] Mariño G., Ugalde A.P., Salvador-Montoliu N., Varela I., Quirós P.M., Cadiñanos J., van der Pluijm I., Freije J.M.P., López-Otín C. (2008). Premature aging in mice activates a systemic metabolic response involving autophagy induction. Hum. Mol. Genet..

[74] Ben-Zvi A., Miller E.A., Morimoto R.I. (2009). Collapse of proteostasis represents an early molecular event in *Caenorhabditis elegans* aging. Proc. Natl. Acad. Sci. USA.

[75] Powers E.T., Morimoto R.I., Dillin A., Kelly J.W., Balch W.E. (2009). Biological and Chemical Approaches to Diseases of Proteostasis Deficiency. Annu. Rev. Biochem..

[76] Cohen E., Dillin A. (2008). The insulin paradox: Aging, proteotoxicity and neurodegeneration. Nat. Rev. Neurosci..

[77] Kaushik S., Cuervo A.M. (2015). Proteostasis and aging. Nat. Med..

[78] Ding W.-X., Ni H.-M., Gao W., Yoshimori T., Stolz D.B., Ron D., Yin X.-M. (2007). Linking of autophagy to ubiquitin-proteasome system is important for the regulation of endoplasmic reticulum stress and cell viability. Am. J. Pathol..

[79] Ding W.-X., Ni H.-M., Gao W., Chen X., Kang J.H., Stolz D.B., Liu J., Yin X.-M. (2009). Oncogenic transformation confers a selective susceptibility to the combined suppression of the proteasome and autophagy. Mol. Cancer Ther..

[80] Megalou E.V., Tavernarakis N. (2009). Autophagy in *Caenorhabditis elegans*. Biochim. Biophys. Acta Mol. Cell Res..

[81] Chen X., Yin X.M. (2011). Coordination of autophagy and the proteasome in resolving endoplasmic reticulum stress. Vet. Pathol..

[82] Cuervo A.M., Bergamini E., Brunk U.T., Dröge W., Ffrench M., Terman A. (2014). Autophagy and Aging: The Importance of Maintaining “Clean” Cells. Autophagy.

[83] Kikis E.A., Gidalevitz T., Morimoto R.I. (2010). Protein homeostasis in models of aging and age-related conformational disease. Adv. Exp. Med. Biol..

[84] Basaiawmoit R.V., Rattan S.I.S. (2010). Cellular stress and protein misfolding during aging. Methods Mol. Biol..

[85] David D.C., Ollikainen N., Trinidad J.C., Cary M.P., Burlingame A.L., Kenyon C. (2010). Widespread Protein Aggregation as an Inherent Part of Aging in *C. elegans*. PLoS Biol..

[86] Hardy J., Selkoe D.J. (2002). The amyloid hypothesis of Alzheimer’s disease: Progress and problems on the road to therapeutics. Science.

[87] Bennett M.C. (2005). The role of α-synuclein in neurodegenerative diseases. Pharmacol. Ther..

[88] Galbiati M., Crippa V., Rusmini P., Cristofani R., Cicardi M.E., Giorgetti E., Onesto E., Messi E., Poletti A. (2014). ALS-related misfolded protein management in motor neurons and muscle cells. Neurochem. Int..

[89] Cuervo A.M. (2008). Autophagy and aging: Keeping that old broom working. Trends Genet..

[90] Keller J.N., Dimayuga E., Chen Q., Thorpe J., Gee J., Ding Q. (2004). Autophagy, proteasomes, lipofuscin, and oxidative stress in the aging brain. Int. J. Biochem. Cell Biol..

[91] Hansen M., Chandra A., Mitic L.L., Onken B., Driscoll M., Kenyon C. (2008). A role for autophagy in the extension of lifespan by dietary restriction in *C. elegans*. PLoS Genet..

[92] Meléndez A., Tallóczy Z., Seaman M., Eskelinen E.-L., Hall D.H., Levine B. (2003). Autophagy genes are essential for dauer development and life-span extension in *C. elegans*. Science.

[93] Vellai T., Takacs-Vellai K., Zhang Y., Kovács A.L., Orosz L., Müller F. (2003). Genetics: Influence of TOR kinase on lifespan in *C. elegans*. Nature.

[94] Lapierre L.R., Meléndez A., Hansen M. (2012). Autophagy links lipid metabolism to longevity in *C. elegans*. Autophagy.

[95] Lapierre L.R., Gelino S., Meléndez A., Hansen M. (2011). Autophagy and lipid metabolism coordinately modulate life span in germline-less *C. elegans*. Curr. Biol..

[96] Madeo F., Tavernarakis N., Kroemer G. (2010). Can autophagy promote longevity?. Nat. Cell Biol..

[97] Singh R., Kaushik S., Wang Y., Xiang Y., Novak I., Komatsu M., Tanaka K., Cuervo A.M., Czaja M.J. (2009). Autophagy regulates lipid metabolism. Nature.

[98] Czaja M.J. (2010). Autophagy in health and disease. 2. Regulation of lipid metabolism and storage by autophagy: Pathophysiological implications. Am. J. Physiol. Cell Physiol..

[99] Rodriguez-Rocha H., Garcia-Garcia A., Panayiotidis M.I., Franco R. (2011). DNA damage and autophagy. Mutat. Res..

[100] Buckley K.M., Melikian H.E., Provoda C.J., Waring M.T. (2000). Regulation of neuronal function by protein trafficking: A role for the endosomal pathway. J. Physiol..

[101] Reenstra W.R., Yaar M., Gilchrest B.A. (1996). Aging affects epidermal growth factor receptor phosphorylation and traffic kinetics. Exp. Cell Res..

[102] Deák F. (2014). Neuronal vesicular trafficking and release in age-related cognitive impairment. J. Gerontol. A Biol. Sci. Med. Sci..

[103] Jiang S., Li Y., Zhang X., Bu G., Xu H., Zhang Y.-W. (2014). Trafficking regulation of proteins in Alzheimer’s disease. Mol. Neurodegener..

[104] Hunn B.H. M., Cragg S.J., Bolam J.P., Spillantini M.-G., Wade-Martins R. (2015). Impaired intracellular trafficking defines early Parkinson’s disease. Trends Neurosci..

[105] Huang Y., Thathiah A. (2015). Regulation of neuronal communication by G protein-coupled receptors. FEBS Lett..

[106] Zhang P., Torres K., Liu X., Liu C.-G., Pollock R.E. (2016). An Overview of Chromatin-Regulating Proteins in Cells. Curr. Protein Pept. Sci..

[107] Matsuoka S., Ballif B.A., Smogorzewska A., McDonald E.R., Hurov K.E., Luo J., Bakalarski C.E., Zhao Z., Solimini N., Lerenthal Y. (2007). ATM and ATR Substrate Analysis Reveals Extensive Protein Networks Responsive to DNA Damage. Science.

[108] Lans H., Marteijn J.A., Schumacher B., Hoeijmakers J.H.J., Jansen G., Vermeulen W. (2010). Involvement of Global Genome Repair, Transcription Coupled Repair, and Chromatin Remodeling in UV DNA Damage Response Changes during Development. PLoS Genet..

[109] Feser J., Tyler J. (2011). Chromatin structure as a mediator of aging. FEBS Lett..

[110] Das C., Tyler J.K. (2013). Histone exchange and histone modifications during transcription and aging. Biochim. Biophys. Acta.

[111] Liu B., Yip R.K., Zhou Z. (2012). Chromatin remodeling, DNA damage repair and aging. Curr. Genomics.

[112] Daniilidou M., Koutroumani M., Tsolaki M. (2011). Epigenetic mechanisms in Alzheimer’s disease. Curr. Med. Chem..

[113] Chen X.-F., Zhang Y.-W., Xu H., Bu G. (2013). Transcriptional regulation and its misregulation in Alzheimer’s disease. Mol. Brain.

[114] Ammal Kaidery N., Tarannum S., Thomas B. (2013). Epigenetic landscape of Parkinson’s disease: Emerging role in disease mechanisms and therapeutic modalities. Neurotherapeutics.

[115] Vilchez D., Saez I., Dillin A. (2014). The role of protein clearance mechanisms in organismal ageing and age-related diseases. Nat. Commun..

[116] López-Otín C., Blasco M.A., Partridge L., Serrano M., Kroemer G. (2013). The hallmarks of aging. Cell.

[117] Riedel C.G., Dowen R.H., Lourenco G.F., Kirienko N.V., Heimbucher T., West J.A., Bowman S.K., Kingston R.E., Dillin A., Asara J.M. (2013). DAF-16 employs the chromatin remodeller SWI/SNF to promote stress resistance and longevity. Nat. Cell Biol..

[118] Li J., Ebata A., Dong Y., Rizki G., Iwata T., Lee S.S. (2008). *Caenorhabditis elegans* HCF-1 functions in longevity maintenance as a DAF-16 regulator. PLoS Biol.

[119] Wolff S., Ma H., Burch D., Maciel G.A., Hunter T., Dillin A. (2006). SMK-1, an Essential Regulator of DAF-16-Mediated Longevity. Cell.

